# C.H. Waddington’s differences with the creators of the modern evolutionary synthesis: a tale of two genes

**DOI:** 10.1007/s40656-017-0143-4

**Published:** 2017-08-08

**Authors:** Jonathan B. L. Bard

**Affiliations:** Department of Physiology, Anatomy and Genetics, University of Oxford, 45 Chalbury Road, Oxford, OX2 6UX UK

**Keywords:** Evolution, Genes, Genetics, Systems biology, Waddington, C.H.

## Abstract

In 2011, Peterson suggested that the main reason why C.H. Waddington was essentially ignored by the framers of the modern evolutionary synthesis in the 1950s was because they were Cartesian reductionists and mathematical population geneticists while he was a Whiteheadian organicist and experimental geneticist who worked with *Drosophila*. This paper suggests a further reason that can only be seen now. The former defined genes and their alleles by their selectable phenotypes, essentially the Mendelian view, while Waddington defined a gene through its functional role as determined by genetic analysis, a view that foresaw the modern view that a gene is a DNA sequence with some function. The former were interested in selection, while Waddington focused on variation. The differences between the two views of a gene are briefly considered in the context of systems biology.

Peterson (2011) considers why more attention was not paid by the framers of the Modern Evolutionary Synthesis to C.H Waddington, and points to their different philosophical and experimental perspectives. His view is that the former were essentially reductionist Cartesians and population geneticists while Waddington was a Whiteheadian organicist and experimental geneticist, who mainly worked on development. It is hard to disagree with Peterson’s view, but, with the perspective of time, one can point to another and perhaps more profound reason for their lack of interest in one another, and that is in their different views of a gene.

For evolutionary population geneticists, a gene is still generally seen as a heritable factor underpinning some trait (e.g. colour, altruism, thumb opposability) whose distinct alleles are responsible for variation, with each having a selective value. Such a gene is basically that introduced by Mendel in 1866. Given this definition, together with a model of population genetics, one can predict changes in allele frequency under selection and hence the early stages (only) of evolutionary change. This is the modern evolutionary synthesis.

All other biologists take a view of a gene that comes from the work on molecular biology done in the early 1960s: here, a gene is a length of DNA with some function, with its exact role being determined either by direct experimental analysis or by mutation (typically, it is either an untranscribed regulatory sequence or a transcribed sequence which is usually translated into a protein such as an enzyme, transcription factor or signal). It is generally impossible to assign a selective value to this class of gene or one of its mutants.

Neither Waddington nor the evolutionary population geneticists could of course have articulated these different types of gene in the 1950s. It was however in this context that Waddington (1965) wrote to Needham saying:

I don’t actually think very much of all the ‘mathematical theory of evolution’ stuff which [Haldane] started—it is all ultimately based on the quite fallacious notion that selection coefficients belong to genes, whereas actually they belong to phenotypes, a matter which has profound consequences, as I showed in my ‘genetic assimilation’ experiments (Waddington to J. Needham, 25 March 1965, JN-CUL, M.18 and quoted from Peterson 2011, p. 311). For a modern view on genetic assimilation, see Bard (2017).

The two distinct types of genes have very different roles in mediating evolutionary change. The Mendelian (or trait) gene is key to understanding selection, which operates on phenotypes, while the modern gene, which was Waddington’s gene even in the 1940s, provides the molecular basis of developmental anatomy and, through mutation, of variation. The prime reason for the disconnect between the framers of the modern synthesis and Waddington was that the former were interested in selection while Waddington focused on developmental anatomy and variation. As they were looking at completely different problems involving different types of gene, it is no wonder that they had little sympathy for one another.

Waddington was a pioneer both in his perspective on evolution (Waddington 1975) and in his work on the genetics of development (Waddington 1956). His research on *Drosophila* in Morgan’s laboratory in the late 1930s and early 1940s (e.g. Waddington 1940, 1941; see Bard 2008) was not only the first serious analysis of the genetics of development, but also produced a methodology that first he and then others used widely. This is the use of abnormal mutants to probe normal development, an approach that underpins all of evo-devo. Waddington’s work led to a detailed analysis of wing development in *Drosophila* and the basis of molecular genetics, while it also provided the scientific evidence that underpinned the metaphor of the epigenetic landscape. Some 50 years later, it was also the methodology that led to the Nobel-prize-winning work on the roles of Hox genes and the molecular basis of apoptosis.

The relationship between the two types of gene is still not clear, but general systems biology approaches suggest that this relationship is unlikely to be either simple or well-defined (Bard 2017). This is because the trait gene is a fairly high-level construct responsible for the details of the anatomical or physiological phenotype, while the modern gene is far more of a nuts-and-bolts item. Figure 1 shows the various levels of role that each play in evolutionary biology, and it is clear that the dividing line is the protein network. It is now known that most major events in developmental anatomy, physiology and biochemistry represent the outputs of complex protein networks (Table 1; many networks are available online at www.sabiosciences.com/pathwaycentral.php). Trait genes mainly reflect the output of one or more networks in particular anatomical contexts; modern genes code for a single component within a network.

**Fig. 1:**
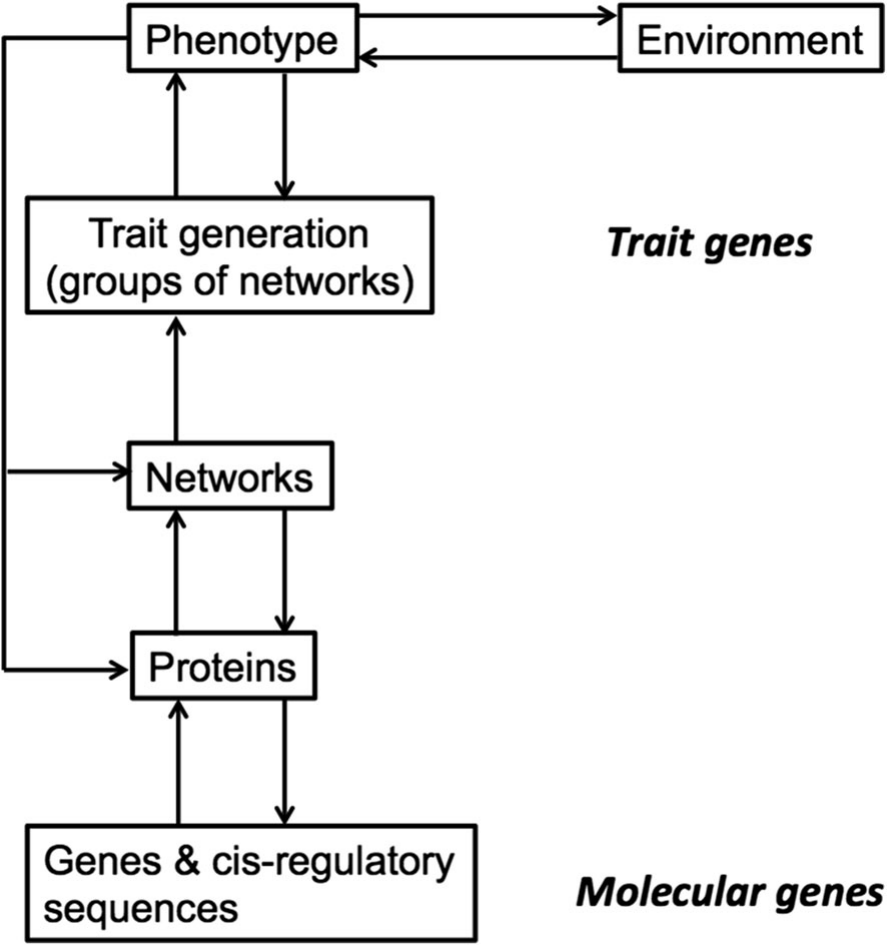
The hierarchy of levels that links genes to the final phenotype of an organism. Note that there is both upwards and downwards feedback. Adapted from Bard (2017)

**Table 1:** Some complex protein networks

Developmental networks	Physiological networks	Biochemical pathways
Signal transduction (6^a^)	Circadian rhythm	Glycolysis
Entering cell proliferation	Heart-beat cycle	Krebs’ cycle
Mitosis	Muscle contraction	Urea cycle
Apoptosis	Neuronal transduction	Photosynthesis
Differentiation (~20^a^)	Immune responses	Polysaccharide metabolism
Morphogenesis (~4^a^)	CAMP pathway	Pentose-phosphate pathway

^a^Number of networks

Waddington could not of course have known all of this, but his 1940s work on the effect of mutations such as *Cubitus interruptus*, which modifies the anatomical details of normal *Drosophila* development and is now known to affect a transcription factor, provided the first indications of the complexity of molecular genetics. This work led to his thinking about the networks that regulate development some twenty years before anyone else (Waddington 1962). These ideas have been mainly been forgotten as molecular facts always supersede clever speculations.

Peterson notes, perhaps sadly, that Waddington was not properly respected in the USA by the founders of the modern synthesis or, incidentally, by its developmental biologists. Things were however very different in the UK where development was treated more conceptually than in the USA. Waddington was also appreciated in the UK as the forerunner of all the work on pattern formation during embryogenesis that was initiated by Wolpert (1969), while the four volumes of *Towards a Theoretical Biology* (1968–1971), the proceedings of four meetings that Waddington organized in Italy, influenced a generation of developmental biologists. As a mark of Waddington’s influence and work, the annual medal of the British Society of Developmental Biology, initiated in 1998, was rightly named after him.
